# Evaluation Criteria for Publishing in Top-Tier Journals in Environmental Health Sciences and Toxicology

**DOI:** 10.1289/ehp.1003280

**Published:** 2011-03-17

**Authors:** Byung-Mu Lee

**Affiliations:** College of Pharmacy, Sungkyunkwan University, Suwon, South Korea

**Keywords:** environmental health sciences, evaluation criteria, peer review, top-tier journals, toxicology

## Abstract

Background: Trying to publish a paper in a top-rated peer-reviewed journal can be a difficult and frustrating experience for authors. It is important that authors understand the general review process before submitting manuscripts for publication.

Objectives: Editors-in-chief and associate editors from top-tier journals such as *Environmental Health Perspectives* (*EHP*), *Toxicological Sciences*, *Journal of Pharmacology and Experimental Therapeutics*, and *Chemical Research in Toxicology* were asked to provide guidance concerning the writing and submission of papers to their journals.

Discussion: The editors reviewed the manuscript review process for their journals, elaborated on the evaluation criteria for reviewing papers, and provided advice for future authors in preparing their papers.

Conclusions: The manuscript submission process was similar for all of the journals with the exception of *EHP* that includes an initial screening in which about two-thirds of submitted papers are returned to the authors without review. The evaluation criteria used by the journals were also similar. Papers that are relevant to the scope of the journal, are innovative, significantly advance the field, are well written, and adhere to the instructions to authors have a higher likelihood of being accepted. The editors advised potential authors to ensure that the topic of the paper is within the scope of the journal, represents an important problem, is carefully prepared according to the instructions to authors, and to seek editorial assistance if English is not the primary language of the authors.

Environmental health sciences is the study of how various biological, physical, or chemical factors influence the health of humans and their surroundings. Research in this area is increasingly multidisciplinary and includes a number of related fields such as toxicology, exposure science, and epidemiology. Environmental health research provides the scientific basis for developing public health strategies to protect human health and approaches to promote a sustainable environment. One of the primary sources of scientific information that is available to researchers and policy makers is the peer-reviewed literature.

Publishing scientific information is also clearly important to researchers in academia, government, and industry. Their careers and their organizations are highly dependent on publishing their findings in the best journals possible. Many scientists have little difficulty designing and conducting experiments, but they tend not to be as successful in getting their work published in a highly rated peer-reviewed journal. Editors of such journals often receive complaints from authors expressing concerns about the peer-review process, especially the quality of the reviews. Many authors wonder why the rejection rate varies so much across journals. The acceptance rate for a journal such as *Environmental Health Perspectives* (*EHP*) is about 20% (Tilson H, personal communication), whereas the acceptance rate for other journals can be as high as 50%. Most editors of journals would agree that the rejection rate for papers from developing countries is higher than for those from developed countries. The reasons for this disparity are not clear but may relate to the fact that most top-tier journals are written in English. This may be problematic for some authors who use English as a second language.

In May 2010, the Korean Society of Toxicology sponsored an international workshop that was held in Seoul, Korea titled “The Way to Top-Tiered Journals in Toxicology.” Editors from *EHP*, *Journal of Pharmacology and Experimental Therapeutics* (*JPET*), *Chemical Research in Toxicology* (*CRT*), and *Toxicological Sciences* (*Toxicol Sci*) were invited to discuss their manuscript review processes and the criteria used to accept or reject papers. These journals receive about 1,000 papers per year and have acceptance rates of 18–50%; their 2009 impact factors ranged from 3.74 to 6.19 (Table 1). The overall goal of the workshop was to provide an understanding of the journal review process in detail in order to improve the quality of papers submitted to scientific journals.

**Table 1:** A brief summary of top journals in environmental health sciences and toxicology, 2009 characteristics.*a*

Table 1. A brief summary of top journals in environmental health sciences and toxicology, 2009 characteristics.*a*								
		Journal						
Characteristic		*EHP*		*JPET*		*Toxicol Sci*		*CRT*
Date publication started		1972		1909		1981		1988
Sponsoring organizations*b*		NIEHS		ASPET		SOT		ACS
No. of papers received		1,286		1,200–1,500		877		—
No. of papers accepted		205		456–570		263		—
Acceptance rate		18%		38%		33%		~ 50%
Publication frequency		Monthly		Monthly		Monthly		Monthly
Impact factor*c*		6.12 (6.19)		4.31 (4.09)		4.44 (4.81)		3.49 (3.74)
Online availability*d*		Open access		Open access		Charge		Charge
**a**Data were provided by the editors of *CRT*,* EHP*,* JPET*,* and Toxicol Sci* in 2009. **b**American Chemical Society, American Society for Pharmacology and Experimental Therapeutics, National Institute of Environmental Health Sciences, and Society of Toxicology. **c**Data were provided by the editors at the time of the workshop in 2010; the values in parentheses represent current impact factors (ISI 2011) . **d**Open access journal articles are freely available, and charge articles are available for a fee.								

## Summary of the Manuscript Review Process

The manuscript review processes for the journals represented at the workshop were similar, with one major difference: *EHP* uses an initial screening process where approximately two-thirds of the papers received are returned to the authors without review. Once a paper has passed this screening process, *EHP* uses a group of consulting editors to decide if the paper is within the scope of the journal and to provide an initial evaluation of the paper for innovativeness, scientific quality, environmental relevance, and clarity. Adherence to the instructions for preparing the manuscript, such as word limits, is also assessed at this time. For all journals represented at the workshop, papers selected for review are assigned to an editor or associate editor, who contacts the peer reviewers and manages the actual review of the paper. These editors usually make a recommendation to the editor-in-chief concerning the acceptability of a manuscript. Most journals have a board of associate editors and an editorial review board, composed of leading scientists with established reputations in the discipline covered in the paper, to assist in the peer-review process. These scientists are intricately involved in the review process, and the quality of the published papers depends heavily on their input.

## Summary of Evaluation Criteria

The evaluation criteria applied by the various journals were also remarkably similar.

*Scope of the journal.* For *EHP*, it is important that the research be relevant to human health. Toxicology studies involving *in vitro* or animal models also must be pertinent to human health. If the study is mechanistically oriented, the end points or indicators must be relevant in humans. If the study concerns wildlife or the environment, the relationship to human health or human activities must be clear. *EHP* has a history of publishing papers concerning the effects of environmentally relevant chemicals (e.g., air pollutants, flame retardants, dioxins, and polychlorinated biphenyls, bisphenol A) on human health or susceptible populations such as children or the elderly. Much of the work published in *EHP* is multidisciplinary. *EHP* publishes several different types of peer-reviewed papers, including commentaries, reviews, and research articles. It also publishes papers that discuss case studies of patients or communities with a clearly established link to environmental exposures and health. The journal welcomes papers related to issues of environmental health policy or risk assessment (EHP 2010). Letters to the editor and editorials are not peer reviewed but are subject to editing and review by *EHP* editors.

*Toxicol Sci* deals with all areas of toxicology, including descriptive, mechanistic, interpretative, translational, and theoretical studies. *Toxicol Sci* is interested in statistical or mechanism-based approaches to risk assessment and new methods in toxicology. This journal covers original research, timely reviews, forums, policy, and regulatory and controversial issues (Toxicol Sci 2010).

*JPET* covers a broad field involving interactions between chemicals and biological systems from pharmacological and toxicological perspectives. *JPET* publishes papers related to mechanistic, molecular, cellular, animal, and human investigations. Papers submitted to *JPET* should take a mechanistic approach to be of interest to a broad range of toxicologists and pharmacologists (JPET 2010).

*CRT* encourages research articles that address all aspects of the chemical bases of toxic responses. It emphasizes rigorous chemical standards and encourages the application of modern techniques of chemical analysis to toxicity mechanisms. *CRT* publishes peer-reviewed articles, reviews, perspectives, communications, chemical profiles, and letters to the editor (CRT 2010).

*Innovation.* All of the journals stressed the importance of demonstrating the novelty of the findings. Authors must make it clear in the manuscript, particularly in the “Discussion,” how the research introduces a new or novel concept or how the current work differs from research that has been previously published. Failure to do so is one of the most common reasons for rejection. *EHP* considers whether a paper adds to the “weight of the evidence” in a particular field. If the research is an extension of already existing literature, authors should make it clear how the current findings confirm or support previous results in a different study population or over a longer period of time, or confirm findings using a new or different study design, experimental model, data and sample collection method, or analytic technique. At *EHP*, papers have a relatively high likelihood of being rejected without review if they address a study question in a small or poorly characterized study population or use a less reliable or less valid data collection method or analytic approach than in previously published studies.

*Experimental design and methodology.* All top-tier journals are interested in papers that are “well motivated.” In the introduction of their papers, authors should take particular care to describe the scientific rationale for conducting the research. Toxicology journals prefer to see clearly stated hypotheses and experiments designed to address those hypotheses. The “Discussion” should be organized around the hypothesis and how the results of the experiments support the original hypothesis. Top-tier journals are rarely interested in descriptive papers with little rationale.

Authors of experimental research papers should take special care in developing and explaining the experimental design of the research. A section providing details concerning the statistical analyses is also important. Consulting a statistician for advice about the design and statistical analysis of the data before starting the research is highly recommended. In addition, some authors do not use the most up-to-date methods or procedures that are relevant to the experiments. Often papers are rejected because the analytical procedures are insensitive and would probably not be able to detect subtle changes at relatively low exposure levels or concentrations. Authors should keep in mind that methods developed for use in one species may not work in another species.

Sometimes emphasis differs among environmental health science journals such as *EHP* and toxicology journals regarding exposure levels or concentrations in laboratory animal or *in vitro* studies. Toxicology studies frequently investigate dose-related effects of chemicals in animal models or *in vitro* models. Often, mechanistically based studies employ relatively high doses or concentrations that may not be environmentally relevant. Environmental health science journals are interested in experimental dose-related effect studies but emphasize that the doses or concentrations should be environmentally relevant.

In reporting the results of experimental research, it is usually a good strategy to present the findings in the sequence that the experiments are described in the methods section. The narrative in the text should present the data in a logical and straightforward manner. It is crucial that the narrative accurately describe the results portrayed in the tables and figures. Tables and figures should be constructed so that they are easy to read and compare the information with the description in the text. There is also an increasing tendency for authors to use several panels in a single figure, perhaps because it is easier to see all the data in one place, or because authors are trying to reduce the total number of figures in the paper. Regardless of the reason, if the reviewer cannot understand or see the images in the figures, they are unlikely to respond to the paper in a positive manner.

One of the major reasons that journals reject papers is the poor quality of the data. It is difficult to provide a compelling explanation of the results and a supportable set of conclusions if there is high variability, missing or inadequate controls, or internal inconsistencies in the data set.

Many journals do not place a high priority on studies with negative results. Editors and reviewers reason that the negative results could be based on improper experimental design, inappropriate doses or exposure conditions, high variability in the data, or a small number of subjects. In some cases, however, reporting negative data can be as important as a study with positive data. A well-designed and executed negative study can provide valuable insight for future research and, in some cases, demonstrate that a dominant theory or paradigm may be erroneous. The burden of demonstrating the quality of a negative study is clearly on the authors.

Finally, it is crucial to maintain a degree of objectivity in reporting and discussing the data. Papers are often rejected because the authors overinterpret their results, attributing causality where there is only association or discussing trends as if they were profound and highly significant findings. The “Discussion” should consider and discuss alternative explanations for the findings. Some journals, including *EHP*, expect authors to include a discussion of the potential limitations of the study.

*Impact.* All journals are concerned about the potential impact of the research on their respective fields. One way to measure influence is the impact factor, which is the total number of citations divided by the number of papers published over a certain period of time (usually 2 or 5 years). The higher the impact factor, the greater the presumed impact of the journal. Innovative papers reporting novel findings or methods, or certain types of papers, such as review articles, are often highly cited and are therefore of interest to most journals. Editors and reviewers often look to see if the paper is a “least publishable unit,” a paper that contains all of the parts of a paper but in fact reports little new information or is a derivative of something already published elsewhere. Editors and reviewers frequently have to make a judgment call about the potential impact of submitted papers. Papers that are well motivated and designed and that report novel results are rarely rejected.

*Instructions to authors.* Every journal provides detailed instructions for preparing manuscripts, usually online. Instructions for acceptable length, number of words or page limits, format for text and references, preparation of tables and references, and the use of supplemental materials should be followed closely. Papers are sometimes returned to authors for failing to follow the instructions to authors.

For assistance in writing manuscripts, the authors might consult style guides by the American Medical Association (2007) or the American Chemical Society (Coghill and Garson 2006), or *The Elements of Style* (Strunk and White 1999). The International Committee of Medical Journal Editors (2010) has published guidance for writing and editing biomedical publications. Day and Gastel (2006) have published a book on how to write and publish a scientific paper. Zeiger (2000) has published a step-by-step guide to writing a biomedical manuscript and provides lots of examples and exercises for both nonnative and native English speakers. Claxton (2005a, 2005b) has reviewed the issues of scientific authorship and scientific fraud. In addition, Claxton (2007) reviews the conflict of interest and bias for toxicologists.

Frequently, papers are written by authors for whom English is a second language. In addition to making a paper difficult to read by the larger scientific community, imprecise phrasing or improper word use can lead to misinterpretations of the authors’ intent or meaning. In such cases, journals often send the paper back to authors with instructions to seek editorial assistance in preparing the manuscript. Professional editorial companies or consultants may be used but can be costly. Groups such as AuthorAid (2011) provide online services for networking, mentoring, resources, and training for researchers, especially those in developing countries.

*Ethics.* Top-rated journals require assurances that animals used in research have been treated humanely and in accordance with university or institute guidelines to alleviate pain and suffering. If humans are involved, authors must indicate that the research was approved by an appropriate institutional review panel, board, or equivalent. Research involving human subjects must comply with all laws and regulations in the country where the work is being conducted, including requirements for obtaining informed consent.

Journals are interested in publishing new work, so contributions must be original works and not published previously or simultaneously submitted to another journal. Reproduction of materials from previously published materials is sometimes allowed, for example, in review articles or commentaries, but authors must document that they have acquired permission or resolved all copyright issues. Failure to do so could result in rejection of the paper.

One of the most difficult issues for journals to deal with is actual or perceived competing financial interests. The concern is that the design, execution, or reporting of results could be skewed or biased by individuals or groups that support the research financially. There is concern that a controlling authority may interfere with the free flow of information in the scientific community. Some journals, including as *EHP*, have developed a policy requiring authors to declare any actual or potential financial conflicts of interest before the paper will be reviewed. Disclosure of competing interests does not imply that the research itself is questionable, and the decision to accept a paper is not based solely on a declaration concerning a competing interest.

Journals are increasingly faced with the problem of scientific misconduct, including data fabrication or plagiarism. Authors should also be aware that most journals routinely use software to detect plagiarism, and some journals screen all papers for plagiarism—the use of previously published ideas or results without appropriate attribution. Some authors may not be aware that recycling large segments from introduction and discussion sections of previously published papers by the same laboratory or group is a form of self-plagiarism. Journals may return papers to the authors without review if a significant amount of self-plagiarism is detected. Authors also should know that documented instances of plagiarism and allegations of data fabrication may be brought to the attention of the host university or organization.

## Summary and Recommendations to Potential Authors

Publishing research in peer-reviewed scientific journals can be a difficult and frustrating experience, especially for younger scientists. Although some graduate schools offer courses in scientific writing, most students learn how to write from mentors. Students should look for opportunities to enroll in courses offering scientific writing and, perhaps, look for mentors willing to take the time and effort to train their students in this important job skill. Universities should be more proactive in offering scientific writing as part of the core curriculum. Mentors should consider teaching students how to write to be as important as teaching them how to design and conduct research.

In many respects, journal editors often function as mentors to authors who submit their papers. Based on their collective experiences, the following general recommendations should be considered by future authors.

*Make sure that the topic is within the scope of the journal.* Some good toxicological papers may not be acceptable in an environmental health science journal. Epidemiological or observational studies in humans have a low probability of being accepted in a toxicology journal. Ecological studies dealing strictly with wildlife or environmental observations are generally not suitable for toxicological or environmental health journals. Learn the difference between public health research and environmental health research. Go to the journal you are interested in and examine the types of papers that have been published in the past.

*Pick an important problem to study.* Journals are not interested in “me too” papers that do not advance the field or discipline. Make sure that the paper can demonstrate the innovativeness of the findings and the impact of the results. One of the major reasons for rejecting a paper is that it provides a limited advance beyond work already published.

*Be extremely careful in preparing the manuscript.* Sloppy, poorly written papers are almost never accepted. Take care to provide the proper motivation or rationale for the study. Take time to explain the methods, remembering that this section is intended to help others understand what you did and, possibly, reproduce your work. Remember that one major reason for rejecting a paper is that the data are of poor quality. For example, experimental studies with highly variable data sets or that lack appropriate controls are rarely accepted. Write the results section in clear terms, and make it easy to refer the findings to the methods section. Tables and figures should be helpful aids in understanding the findings, not a maze to be navigated. The “Discussion” should address the rationale or hypothesis presented in the introduction section and explain the impact of the results by placing the new data within the context of the literature in the field. Do not overinterpret the data. Consider alternative explanations and discuss possible limitations associated with the study.

*Read the instructions to authors before preparing the manuscript, and follow them closely.* Many papers are returned to authors because of a major failure to follow instructions.

*Scientific writing is in English.* It is important that skills be developed to express thoughts and concepts in English so that the larger scientific community can understand them. Seek editorial assistance before submitting a paper to a journal.
